# Serum HER2 Is a Potential Surrogate for Tissue HER2 Status in Gastric Cancer: A Systematic Review and Meta-Analysis

**DOI:** 10.1371/journal.pone.0136322

**Published:** 2015-08-20

**Authors:** Kecheng Zhang, Jianxin Cui, Hongqing Xi, Shibo Bian, Liangang Ma, Weisong Shen, Jiyang Li, Ning Wang, Bo Wei, Lin Chen

**Affiliations:** Department of General Surgery, Chinese People’s Liberation Army General Hospital, Beijing, China; Fondazione IRCCS Istituto Nazionale dei Tumori, ITALY

## Abstract

Determining the expression level of human epidermal growth factor receptor 2 (HER2) in tumor tissue is of great importance for personalized therapy in gastric cancer. Although several studies have investigated whether serum HER2 can serve as a surrogate for tissue HER2 status, results have been inconsistent. We therefore performed a meta-analysis of published clinical studies in an attempt to address this problem. PubMed, Embase, Web of Science, the Cochrane Library and Science Direct were queried for eligible studies that could provide sufficient data to construct 2 × 2 contingency tables. The quality of the studies included in the meta-analysis was assessed in accordance with the revised Quality Assessment of Diagnostic Accuracy Studies (QUADAS-2) criteria. The pooled sensitivity, specificity and diagnostic odds ratio (DOR) were calculated for the eligible studies. The summary receiver operating characteristic (SROC) curve was constructed and the area under the SROC (AUSROC) was used to evaluate overall diagnostic performance. Eight studies comprising a total of 1170 participants were included in our meta-analysis. The pooled sensitivity, specificity and DOR were 0.39 (95% CI: 0.21–0.61), 0.98 (95% CI: 0.87–1.00), and 27 (95% CI: 9–81), respectively. The AUSROC was 0.77 (95% CI: 0.73–0.80) and Deeks funnel plot suggested the absence of publication bias (p = 0.91). Meta-regression analysis indicated that threshold effect was the main source of heterogeneity. Assays for evaluating serum HER2 levels are highly specific and demonstrate moderate diagnostic performance for HER2 tissue status in gastric cancer.

## Introduction

The past few years have witnessed groundbreaking advances in the application of personalized treatment based on a patient’s genetic background. For gastric cancer, the 2010 phase III Trastuzumab for Gastric Cancer (ToGA) clinical trial demonstrated the efficacy and safety of trastuzumab in the management of human epidermal growth factor receptor 2 (HER2)-positive advanced gastric or gastroesophageal junction (GEJ) cancer [1]. HER2 is encoded by the *HER2/neu* oncogene and belongs to the HER family of tyrosine kinase receptors. These receptors play a central role in the regulation of various cellular processes including growth, survival and migration, and represent candidate molecular therapeutic targets in a range of cancer types [2]. HER2 overexpression is observed in 6% to 35% of gastric cancers [3] and the relationship between this overexpression and patient prognosis remains controversial [4–6]. HER2-positive patients with gastric cancer qualify for trastuzumab-based therapy and, therefore, determination of HER2 status in patient tumor tissue is of great importance prior to the administration of anti-HER2 targeted treatment.

In current clinical practice, tissue samples obtained by surgery or biopsy are primarily used to evaluate a patient’s HER2 status following analysis by immunohistochemistry (IHC) or fluorescence in situ hybridization (FISH) [7, 8]. However, these invasive procedures cannot be performed repeatedly to check for dynamic changes in HER2 status during patient treatment or follow-up because they represent impractical methods for monitoring treatment response. Furthermore, complications associated with biopsy can be encountered [9]. Additionally, conflicting results from IHC or FISH analysis, which may arise because of variations in specimen processing procedures between different laboratories, the existence of non-standardized assays and scoring systems, or the inherent heterogeneity within tumor cells, can also prove problematic [10, 11]. In view of such potential drawbacks, investigators have become increasingly interested in developing more convenient, reproducible and less invasive detection methods for identifying HER2-positive patients.

The expression level of serum HER2 represents a noninvasive biomarker that could supplement current HER2 testing. Studies have revealed a relatively high concordance rate between elevated serum HER2 levels and positive HER2 status in tumor tissues [12, 13]. It has also been reported that serum HER2 could potentially be used to monitor breast cancer relapse [14]. Compared with biopsy, serum HER2 analysis provides a more reproducible and suitable option as a general screening test for the characterization of a cancer patient’s genetic profile, and would therefore greatly benefit the field of targeted cancer therapy. With respect to gastric cancer, several clinical centers have investigated correlations between serum HER2 and clinicopathological characteristics in addition to the tissue HER2 status of patients [15–17]. Changes in serum HER2 levels during chemotherapy have been reported to correlate with response to chemotherapy in patients with HER2-positive tumors [18]. Nonetheless, these studies differ in many aspects such as cohort size, sampling time, tumor-node-metastasis (TNM) stage, and cut-off values, all of which complicate the ability to draw definitive conclusions.

In this study we therefore conducted a meta-analysis to investigate the diagnostic accuracy of serum HER2 for determining tumor HER2 status. To the best of our knowledge, this work has not been performed previously.

## Methods

The meta-analysis was conducted in accordance with the standard guidelines for systematic reviews of diagnostic test accuracy [19] and used the Preferred Reporting Items for Systematic Reviews and Meta-Analyses (PRISMA) statement [20] as the template for reporting (S1 PRISMA Checklist).

### Search strategy

PubMed, Embase, Web of Science, the Cochrane Library and Science Direct electronic databases were searched for entries recorded from the time of database inception to 17 January, 2015. The elements of the following three categories were applied in various combinations when interrogating databases: (“serum” or “soluble”) and (“human epidermal growth factor receptor 2” or “HER2” or “c-erbB-2”) and (“gastric cancer” or “stomach neoplasm” or “stomach tumor”). English language restriction was applied to all searches. Review articles and reference lists were also manually screened for relevant studies.

### Study selection

Two investigators independently reviewed the abstract and title of each publication to identify those articles that were likely to assess the diagnostic value of serum HER2 in gastric cancer. When such articles were identified full-texts were further screened to evaluate study relevance. Eligible studies were selected according to the following inclusion criteria: i) expression levels of both serum and tissue HER2 were detected in gastric cancer patients; ii) the diagnosis of gastric cancer was confirmed by histopathological or cytological examination; iii) sufficient data was available to construct a 2 × 2 contingency table. Exclusion criteria were as follows: i) duplicates; ii) conference abstracts, comments, letters and animal trials; iii) insufficient data to calculate for sensitivity and specificity, i.e., false and true positives and negatives were not available. When there was uncertainty regarding eligibility, a discussion would be held between the two investigators and a senior investigator until consensus was reached.

### Data extraction and quality assessment

Two investigators independently extracted data from each study, such as surname of first author, year of publication, country of origin, enrolled participants, TNM stage, major tumor subtype (Lauren’s classification), methods of serum HER2 detection, the cut-off value, and the numbers of true positives (TP), false positives (FP), false negatives (FN) and true negatives (TN). When different cut-off values were used in the same study, the pre-specified value and the associated sensitivity and specificity were extracted [16, 21].

The methodological quality of each eligible studies was assessed by means of the revised Quality Assessment of Diagnostic Accuracy Studies (QUADAS-2) criteria [22], which consist of four key domains that discuss patient selection, index test, reference standard and flow and timing. Each domain deals with the risk of bias while the first three domains also deal with concerns regarding applicability. Signaling questions are included to assist judgment in terms of risk of bias and concerns with applicability. Questions are answered as “yes”, “no” or “unclear” and are phrased such that “yes” implies low risk of bias/concerns and “no” implies high risk of bias/concerns.

### Statistical analysis

A 2 × 2 table was constructed to calculate sensitivity, specificity, positive likelihood ratios (PLR), negative likelihood ratios (NLR) and the diagnostic odds ratio (DOR) by using bivariate regression models, as recommended by the Cochrane Diagnostic Test Accuracy Working Group [19]. The bivariate model takes into account the potential trade-off effect between sensitivity and specificity [23]. The PLR is calculated as follows: sensitivity/(1−specificity) while the NLR is calculated as follows: (1−sensitivity)/specificity. Generally, a PLR > 5.0 and a NLR < 0.2 were considered clinically useful [24, 25]. DOR is a prevalence-independent indicator that combines sensitivity and specificity, and is calculated as: PLR/NLR [26]. The summary receiver operating characteristic (SROC) curve was constructed and area under the SROC (AUSROC) calculated.

The *I* ^*2*^ statistic [27], which describes the percentage of the total variation across studies that is attributable to heterogeneity rather than chance, was used to assess between-study heterogeneity, with a value exceeding 50% indicating the existence of significant heterogeneity. The *I* ^*2*^ statistic is calculated as follows: 100%×(Q-df)/Q, where Q is Cochran’s heterogeneity statistic and df is degrees of freedom. The value of *I* ^*2*^ ranges from 0–100%, with 0% implying no observed heterogeneity, and larger values indicating increasing heterogeneity. Heterogeneity caused by the threshold effect was evaluated by Spearman’s correlation coefficient as described previously [28–30]. Univariable and multivariable meta-regression analyses were performed to detect the amount of non-threshold effect heterogeneity between studies. Deeks funnel plot was applied to evaluate publication bias and a significance level of α = 0.05 was used to determine statistical significance [31].

All analyses were conducted using Stata software (version 12.0, StataCorp, College Station, Texas USA).

## Results

**Fig 1:**
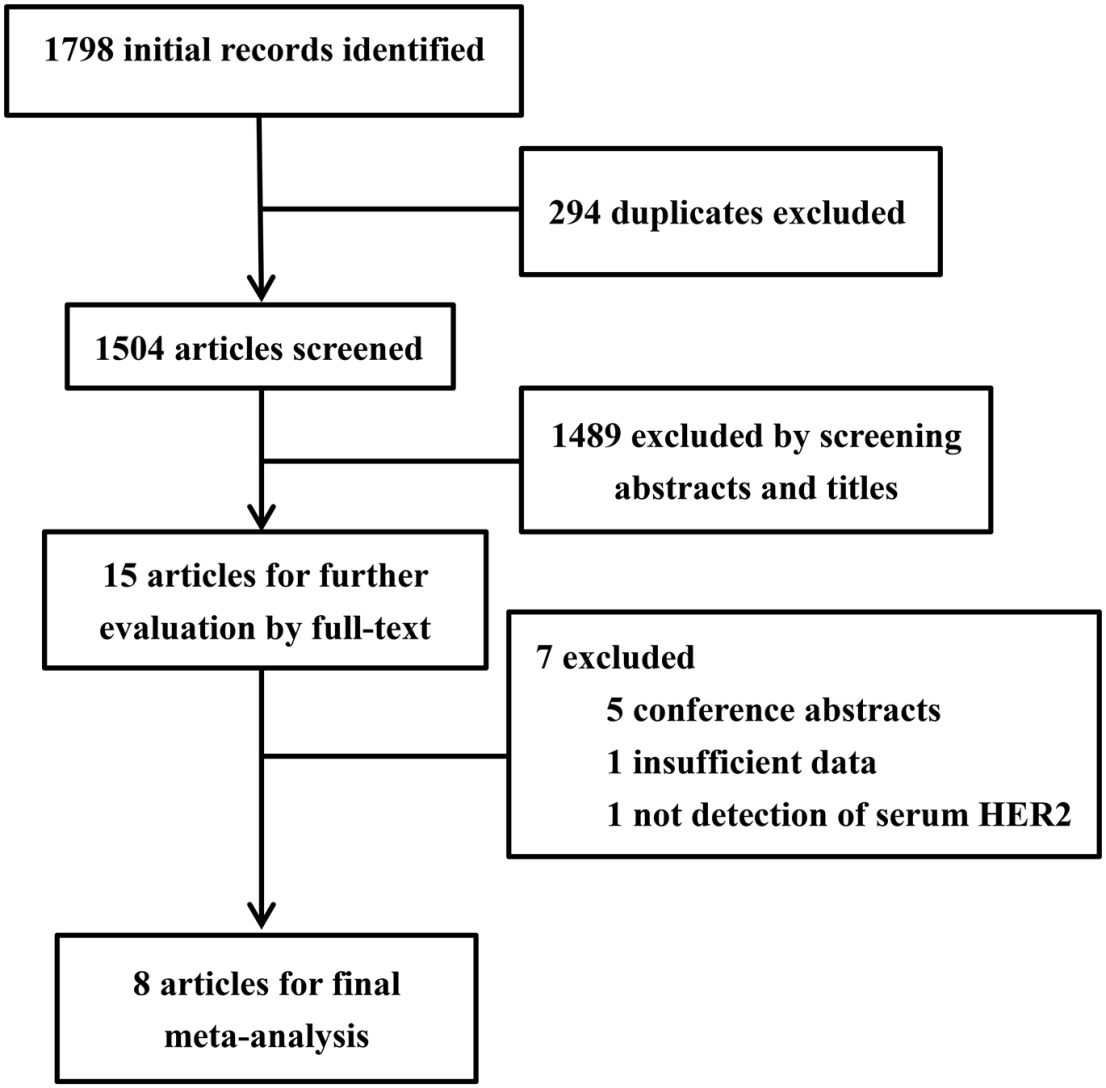
Flowchart of the selection procedure for eligible studies.

After excluding duplications, the initial search yielded 1504 records, of which 15 articles were considered potentially suitable for inclusion in the meta-analysis. Following a review of full-text articles, a further seven were excluded [32–38], leaving a total of eight articles for the pooled analysis [15–18, 21, 39–41]. A flowchart summarizing the selection procedure for study eligibility is shown in Fig 1.

A total of 1170 patients with gastric cancer were included in the meta-analysis with the median patient age among studies ranging from 53 to 71 years. Four studies were conducted retrospectively manner, while the remaining did not specify the design. Seven studies took blood samples prior to any treatment and one study took samples after treatment. The prevalence of HER2 overexpression in gastric cancer patients across the studies ranged from 6.7% to 61.6%. The optimal cut-off value was pre-specified in five of the studies. In these studies a serum HER2 concentration of 15.0 ng/ml (recommended by the Food and Drug Administration for breast cancer) or 15.2 ng/ml (recommended in the manufacturer’s instructions for the relevant commercial kit) was used. The remaining three studies used the concentration of serum HER2 of healthy control individuals as the cut-off point. To determine serum HER2 levels, a chemiluminescence immunoassays (CLIA) was used in five of the studies and an enzyme-linked immunosorbent assay (ELISA) was used in the remaining three studies. Individual study characteristics are summarized in Table 1.

**Table 1:** Characteristics of studies included in the meta-analysis.

Study year	Country	Participants (M/F)	Study design	Median age (range, yrs)	TNM stage	Major location	Lauren’s classification	Sampling time	Prevalence of HER2 overexpression	Cut-off value	Detection method	Assay platform	TP	FP	FN	TN
**Oyama 2015**	Japan	98/42	R	66 (27–86)	I–IV	N/A	Diffuse	Before treatment	16.7%	15.2 ng/ml	CLIA	N/A	9	1	16	124
**Dai 2013**	China	135/84	R	53 (27–77)	Advanced	Non-GEJ	Diffuse	After treatment	16.9%	15.0 ng/ml	CLIA	ADVIA Centaur System	19	7	18	175
**Peng 2014**	China	102/31	R	59 (24–79)	Advanced	Non-GEJ	Intestinal	Before treatment	32.3%	15.0 ng/ml	CLIA	ADVIA Centaur CP Immunoassay System	17	6	26	84
**Sasaki 2015**	Japan	63/33	R	65.5 (29–84)	Advanced	Non-GEJ	Diffuse	Before treatment	19.8%	15.0 ng/ml	CLIA	ADVIA Centaur XP fully automated analyzer	10	5	9	72
**Kono 2000**	Japan	N/A	N/A	65 (N/A)	I–IV	N/A	N/A	Before treatment	15.8%	1241 HNU/ml	ELISA	Commercial ELISA kit	6	3	3	45
**Takehana 2002**	Japan	243/109	N/A	62.9 (18–90)	I–IV	N/A	N/A	Before treatment	8.5%	3757 HNU/ml	ELISA	Commercial ELISA kit	1	0	10	118
**Narita 2013**	Japan	71/34	N/A	71 (39–89)	I–III	Non-GEJ	N/A	Before treatment	6.7%	15.2 ng/ml	CLIA	ADVIA Centaur automated assays	0	0	7	98
**Li 2013**	China	57/18	N/A	62.4 (35–81)	I–IV	N/A	N/A	Before treatment	61.6%	8.2 ng/ml	ELISA	Commercial ELISA kit	36	15	9	13

*TP* true positive; *FP* false positive; *FN* false negative; *TN* true negative; *N/A* not available; *GEJ* gastroesophageal junction cancer; *CLIA* chemiluminescence immunoassays; *ELISA* enzyme-linked immunosorbent assay; *HNU* human neu-antigen unit; *R* retrospectively

**Fig 2:**
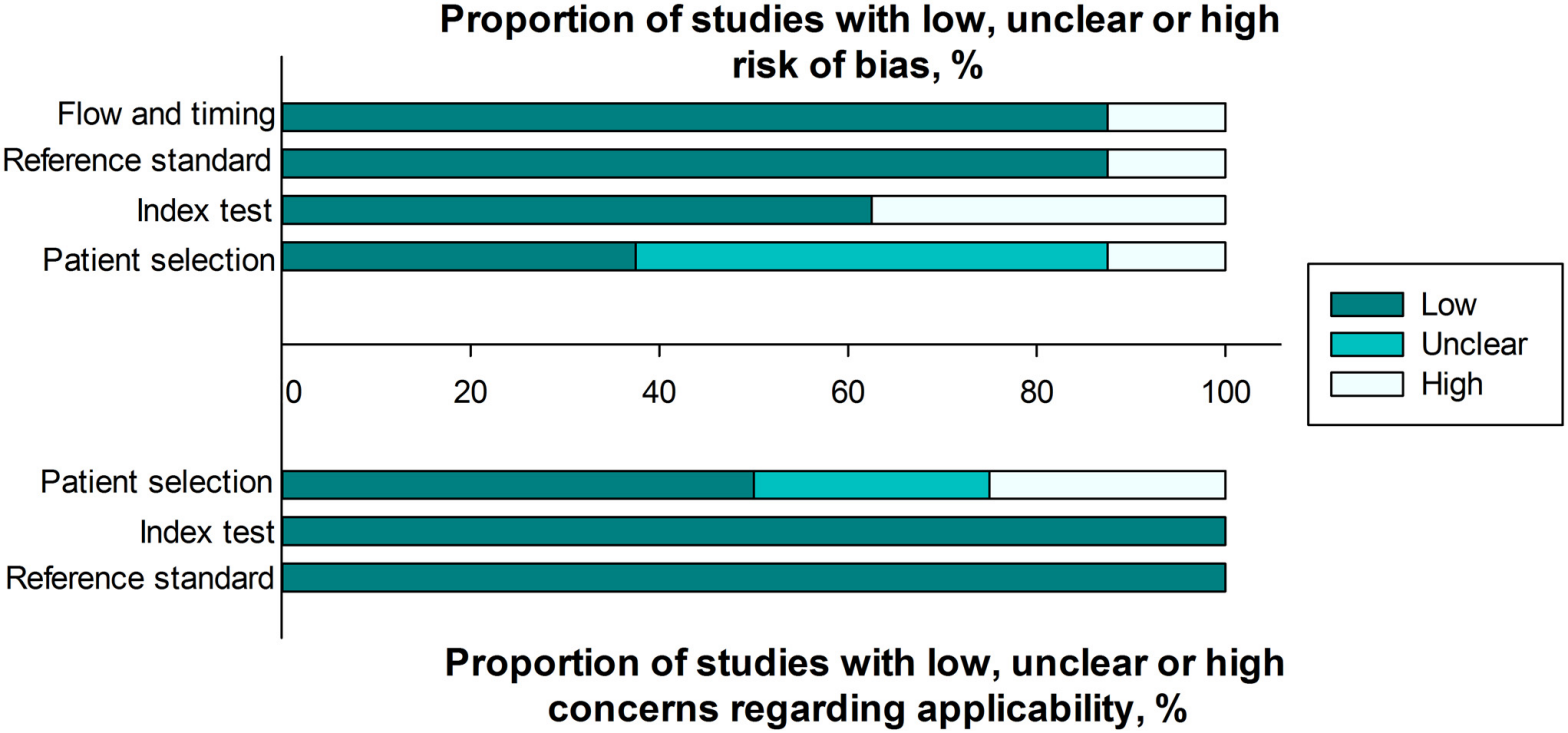
Quality assessments of included studies using the QUADAS-2 tool criteria.

The quality of the included studies was assessed according to QUADAS-2. A graphical summary of the methodological assessment of the included studies is shown in Fig 2.

**Fig 3:**
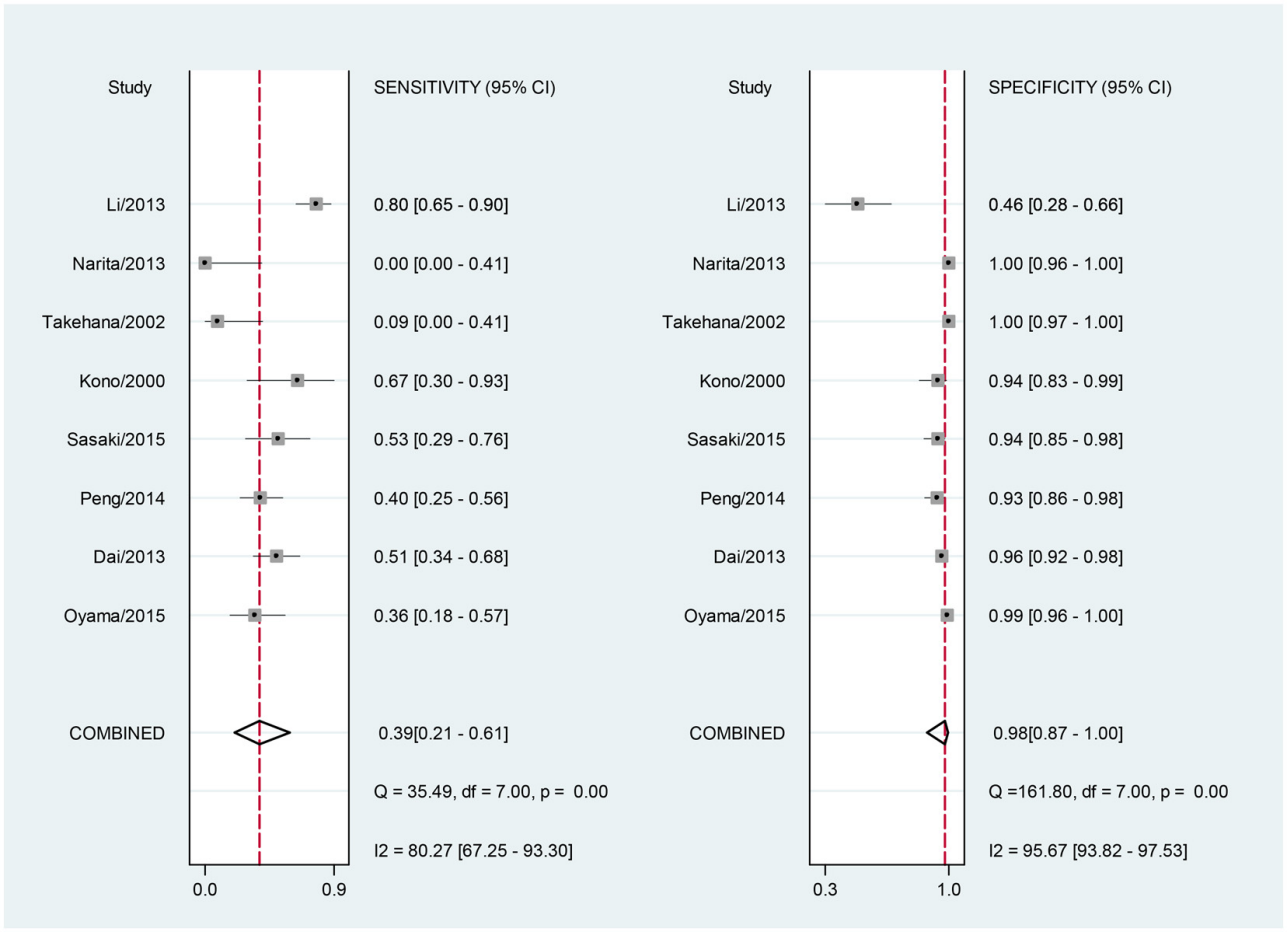
Forrest plot of the sensitivity and specificity of serum HER2 in the diagnosis of HER2-positive patients with gastric cancer with the corresponding heterogeneity statistics.

**Fig 4:**
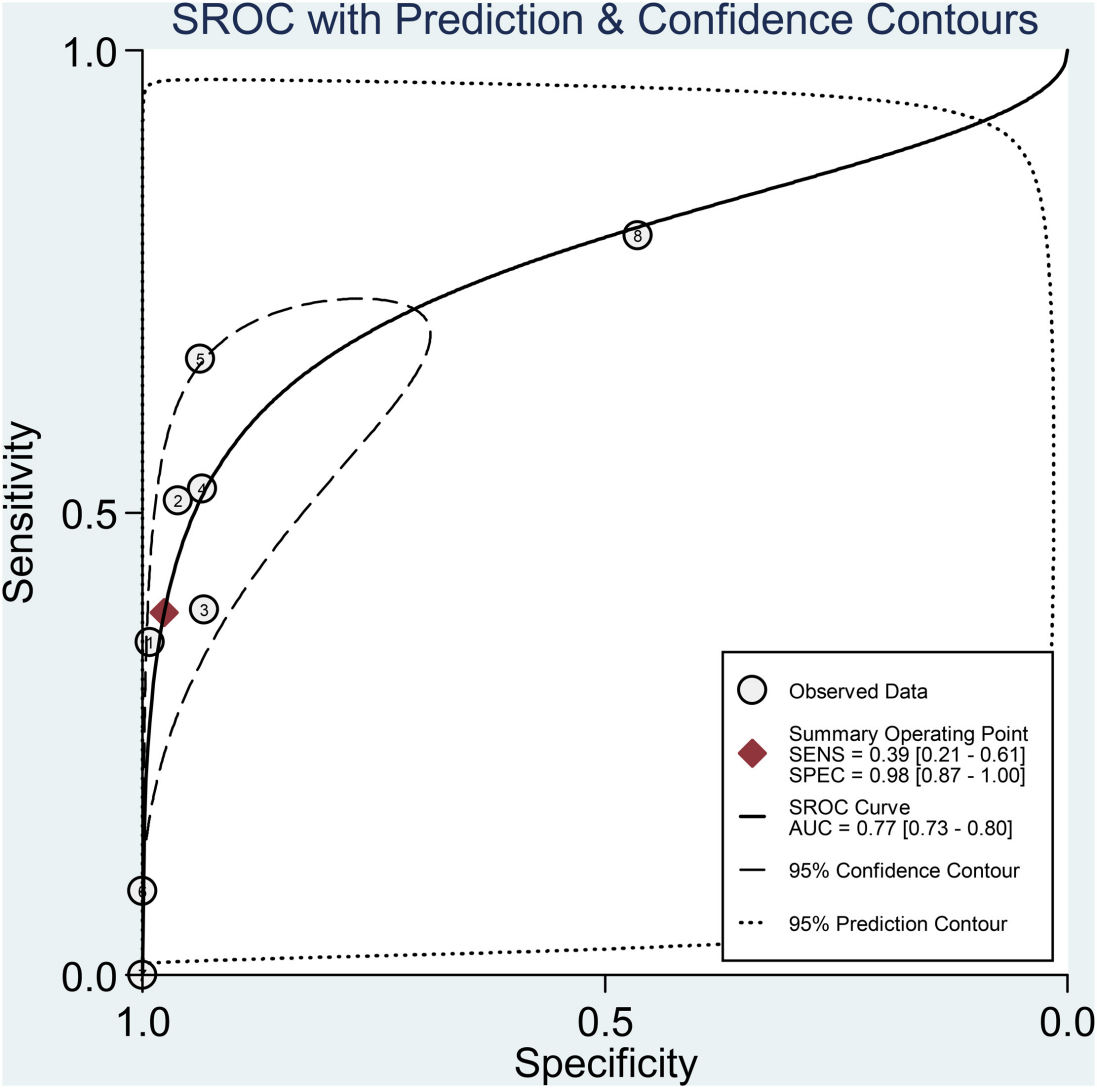
Summary receiver operating characteristic plot for the included studies with the associated 95% confidence region and the 95% prediction region.

**Fig 5:**
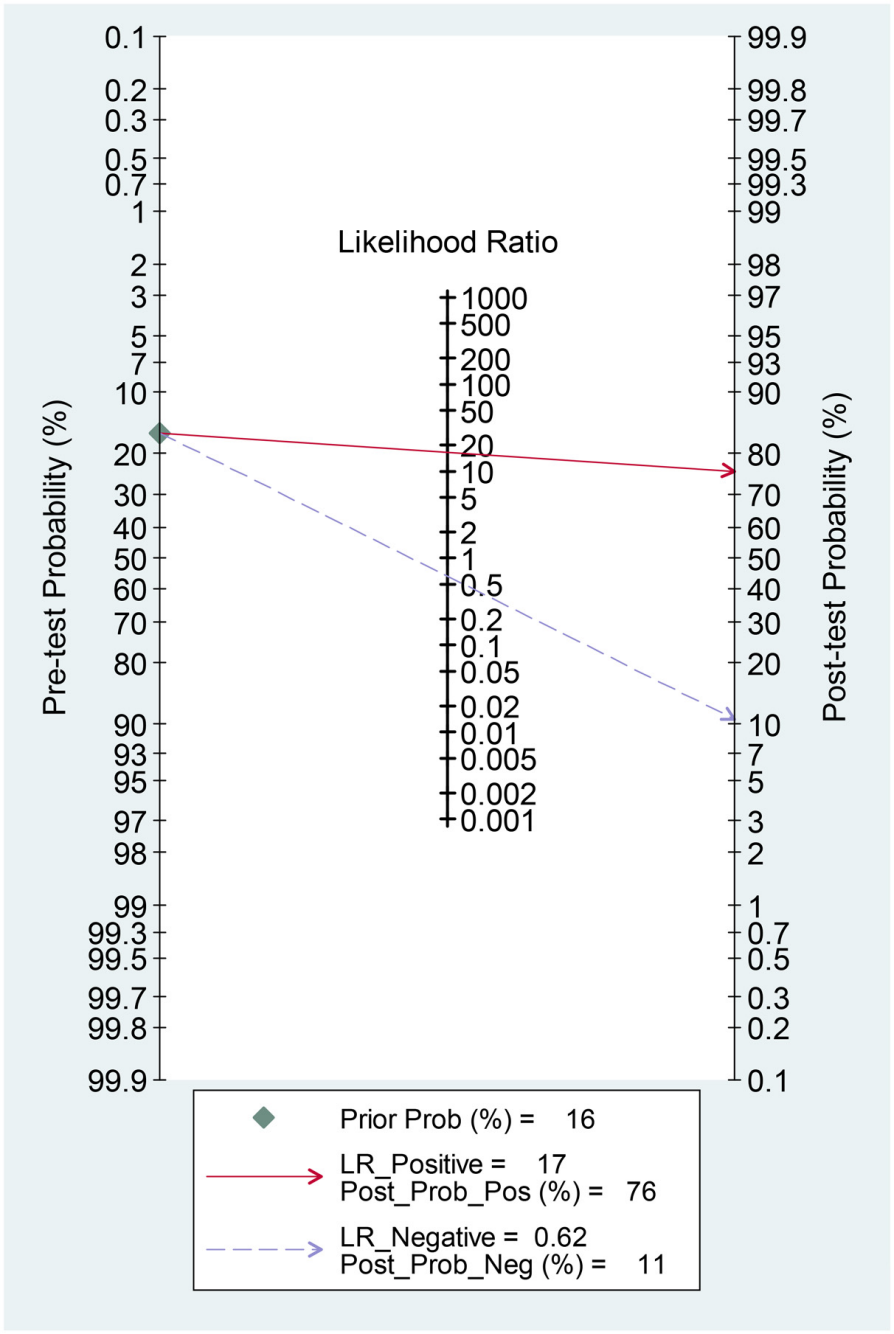
Fagan’s nomogram for the calculation of post-test probabilities with a fixed pre-probability of 16%.

For the included studies, the overall pooled sensitivity was 0.39 (95% CI: 0.21–0.61) and the pooled specificity was 0.98 (95% CI: 0.87–1.00; Fig 3). The pooled PLR and NLR were 16.6 (95% CI: 4.5–61.5) and 0.62 (95% CI: 0.46–0.85), respectively. The area under the SROC was 0.77 (95% CI: 0.73–0.80; Fig 4) and the DOR was 27 (95% CI: 9–81). The results were summarized in Table 2. A Fagan nomogram was used to determine post-test probability based on fixed pre-test probability (Fig 5).

**Table 2:** Summary results of bivariate model analysis and univariable/multivariable meta-regression.

**Bivariate model analysis**							
Sen (95%CI)	*I* ^*2*^_Sen_ (95%CI)	Spe (95%CI)	*I* ^*2*^_Spe_ (95%CI)	PLR (95%CI)	NLR (95%CI)	DOR (95%CI)	AUROC (95%CI)
0.39 (0.21–0.61)	80.27 (67.25–93.30)	0.98 (0.87–1.00)	95.67 (93.82–97.53)	16.6 (4.5–61.5)	0.62 (0.46–0.85)	27 (9–81)	0.77 (0.73–0.80)
**Univariable meta-regression**						**Multivariable meta-regression**	
Parameter	No. of study	Sen (95%CI)	*P*_Sen_	Spe (95%CI)	*P*_Spe_	*I* ^*2*^ (95%CI)	*P*_I_^2^
Stage			0.64		<0.001	0 (0–100)	0.66
I-IV	5	0.35 (0.09–0.62)		0.99 (0.96–1.00)			
III,IV	3	0.47 (0.15–0.79)		0.95 (0.82–1.00)			
Patient size			0.03		0.07	62 (13–100)	0.07
≥100	5	0.28 (0.12–0.45)		0.99 (0.98–1.00)			
<100	3	0.66 (0.45–0.87)		0.85 (0.62–1.00)			
Sampling time			0.55		0.02	0 (0–100)	0.68
Before treatment	7	0.37 (0.14–0.59)		0.98 (0.93–1.00)			
After treatment	1	0.51 (0.00–1.00)		0.96 (0.81–1.00)			
Methods			0.48		0.40	0 (0–100)	0.79
CLIA	5	0.35 (0.11–0.58)		0.98 (0.95–1.00)			
ELISA	3	0.49 (0.15–0.84)		0.96 (0.84–1.00)			
Ethnicity			0.21		<0.001	48 (0–100)	0.15
Japanese	5	0.30 (0.08–0.52)		0.99 (0.98–1.00)			
Chinese	3	0.58 (0.30–0.86)		0.88 (0.65–1.00)			

*Sen* sensitivity; *Spe* specificity; *PLR* positive likelihood ratios; *NLR* negative likelihood ratios; *DOR* diagnostic odds ratios; *AUROC* area under receiver operating curve; *CLIA* chemiluminescence immunoassays; *ELISA* enzyme-linked immunosorbent assay

**Fig 6:**
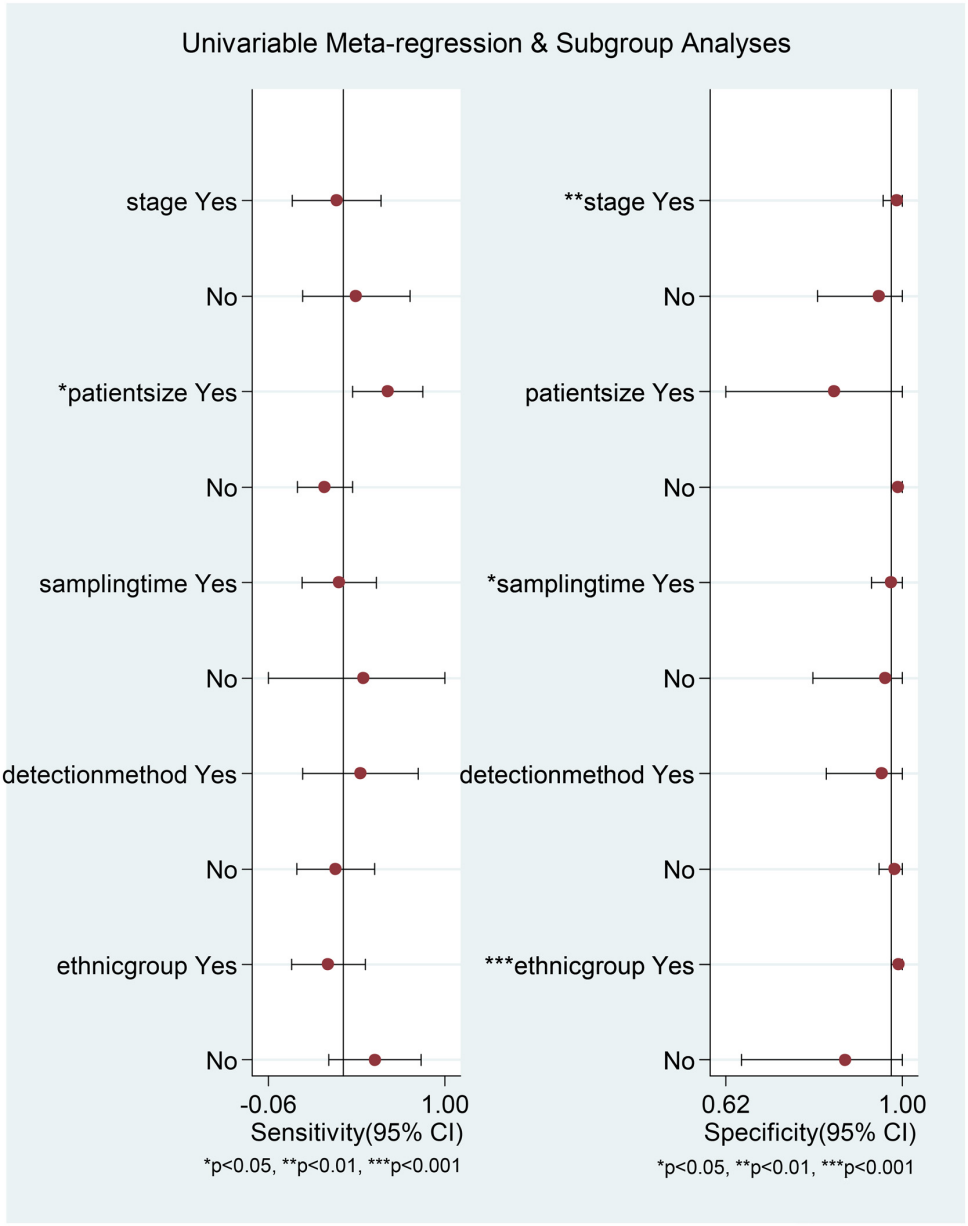
Univariable meta-regression analysis for sensitivity and specificity.

The between-study variability (i.e., heterogeneity) was found to be high for both sensitivity (*I* ^*2*^ = 80.27%) and specificity (*I* ^*2*^ = 95.67%). We next investigated whether there was threshold effect where variations in sensitivity and specificity correlated with differences in the cut-off value of serum HER2 in the included studies. Surprisingly, bivariate model analysis revealed that there was a negative correlation between sensitivity and specificity and that 100% of heterogeneity was likely to be attributed to threshold effect. The Spearman’s correlation coefficient was −1. This type of effect can often result in a shoulder-like curve when sensitivity is plotted against specificity [23], as shown in Fig 4. We also explored other potential sources of heterogeneity by meta-regression analysis. TNM stage, patient group size, sampling time, detection method and ethnicity were used as covariates. Univariable meta-regression analysis revealed that patient group size accounted for the heterogeneity of sensitivity, while TNM stage, sampling time and ethnicity accounted for the heterogeneity of specificity (Fig 6). However, when all of the above covariates were included in a joint analysis model, meta-regression analysis revealed that none were responsible for heterogeneity (p values of 0.66, 0.07, 0.68 0.79 and 0.15 for stage, patient group size, sampling time, detection method and ethnicity, respectively).

**Fig 7:**
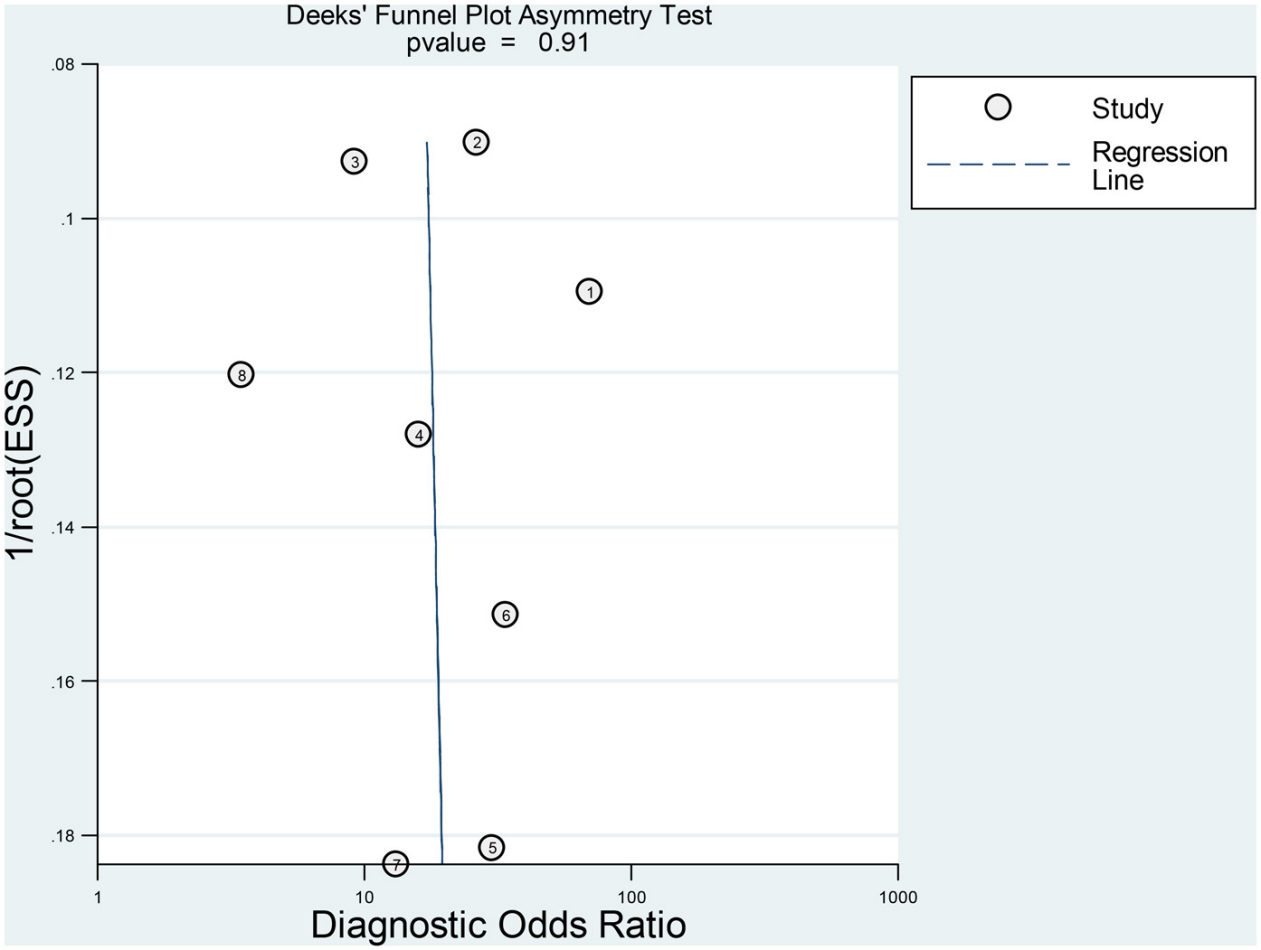
Deeks funnel plot for the evaluation of publication bias.

Finally, Deeks funnel plot indicated the absence of publication bias (p = 0.91) (Fig 7).

## Discussion

HER2-targeted therapy necessitates the determination of HER2 status in patients with cancer. In clinical practice, surgical specimens and biopsy are often used to address this problem. However, surgical specimens are not usually available to patients who do not require tumor resection, and biopsied tissue may provide a false-negative result because of heterogeneous HER2 expression within the tumor [42]. Surgical specimens and biopsied tissue provide only limited information regarding a patient’s HER2 status because they do not permit a real-time picture of HER2 status during disease progression and treatment. In contrast, serum HER2 levels can be examined easily and repeatedly, and in a less invasive manner. Serum HER2 monitoring is also less labor-intensive than surgical techniques and has the potential to be performed by automated assays enabling an objective analysis. Several studies have demonstrated high concordance rates between serum HER2 concentration and tissue HER2 status [12, 43]. In our present meta-analysis, we found that serum HER2 showed high specificity for the detection of tissue HER2 status in gastric cancer.

The AUSROC provides a global estimate of diagnostic performance. According to the suggested guidelines for the interpretation of the AUSROC value [44], serum HER2 had a moderate (0.7 < AUC < 0.9) diagnostic ability to discriminate HER2-positive gastric cancer patients from HER2-negative patients. The DOR can take values between zero and infinity, with higher values indicating better discriminatory test performance. Our pooled analysis yielded a DOR of 27, indicating a moderate diagnostic value of serum HER2 in HER2 tumor status prediction. In clinical settings, it’s important to know how a particular test result predicts risk of abnormality, thus likelihood ratios are often used, which indicate the extent to which a given test would raise or lower the probability of having disease [24]. Fagan’s nomogram is a graphical tool that helps clinicians use test results to estimate a patient’s probability of having a disease [45]. In the nomogram, a line drawn from a patient’s pre-test probability (usually estimated according to local prevalence data and published reports) through the likelihood ratio of the test intersects with the post-test probability, which indicates the patient’s chance of having the disease after the test results are known. In Fig 5, with a pooled PLR of 17 and a hypothetical pre-test probability of 16% (the median prevalence for the eight studies), the use of serum HER2 as a test for HER2-positive tumor diagnosis would raise the post-test probability to 76%, which means that the probability of gastric cancer patients having tissue-HER-2 positive status increases from 16% to 76% in the event of a positive serum-HER2 test. Conversely, with a pooled NLR of 0.62, the post-test probability decreases from 16% to 11% after a negative serum-HER2 test. These findings therefore demonstrate that a HER2 serum test result is of moderate diagnostic value in determining the HER2 status of a patient.

Pooled estimates of sensitivity and specificity were associated with an *I* ^*2*^ of 80.27% and 95.67% respectively, implying the presence of heterogeneity. To our surprise, the bivariate model analysis revealed that all heterogeneity was likely to be the consequence of threshold effect. Threshold effect occurs when the studies included in the meta-analysis use different numerical cut-off values to define a test result as positive [19]. Cut-off values in diagnostic tests represent a major concern as different thresholds can result in variations in sensitivity and specificity. Of the studies included in our meta-analysis, five used cut-off points that were recommended either by the Food and Drug Administration for breast cancer or by the manufacturer of a particular commercial kit used in a study. The remaining three studies used serum HER2 levels calculated for healthy control individuals as a cut-off point and these control concentrations varied between the three studies. Using thresholds that were established for breast cancer may be inappropriate for patients with gastric cancer. Peng et al. have identified the optimal cut-off point of serum HER2 as 10.65 ng/ml for gastric cancer patients, which is lower than the 15.0 ng/ml cut-off point more generally applied [21]. When using this 10.65 ng/ml threshold, ROC analysis yielded a better sensitivity when compared with that obtained for the 15.0 ng/ml threshold (67.4% vs. 40%), while specificity remained unchanged at 78.9%. However, in another study [16], the optimal serum HER2 cut-off value for predicting tissue HER2 status in gastric cancer was found to be 16.35 ng/ml, which exceeds the 15.0 ng/ml threshold. These results suggest that the optimal serum HER2 cut-off value for patients with gastric cancer may differ from that used for breast cancer patients. Kong et al. have even suggested that different ethnicities have different cut-off values and that it may be beneficial to establish cut-off values for each ethnic population [46]. This is an area of research that clearly warrants further investigation.

To explore other potential factors that may account for heterogeneity, we also performed meta-regression analysis. Multivariable meta-regression analysis revealed that TNM stage, patient group size, sampling time, detection method, and ethnicity were not responsible for heterogeneity. This finding further supported our previous result that heterogeneity was likely to be entirely the consequence of threshold effect. In view of the small number of studies included in our analysis, we would interpret the data in a more cautious way and suggest that the relatively small number of eligible studies may compromise the power of test.

It is important to acknowledge the potential limitations of the present meta-analysis. First, despite an in-depth search of several electronic databases, the number of included studies was small. Second, all of the studies were from Asia, which may lead to some inherent bias. Third, we could not determine the ideal cut-off value for the serum HER2 test because the raw data were not provided in the published reports. Large-scale prospective randomized trials are needed to address this problem. Fourth, the quality assessment results shown in Fig 2 indicate that there was a possible risk of bias and concerns regarding applicability in the patient selection procedure in the included studies.

To the best of our knowledge, our study represents the first reported meta-analysis directed at evaluating the clinical utility of serum HER2 in the diagnosis of HER2 tumor status. Our analysis has shown that serum HER2 had high specificity and moderate diagnostic value for distinguishing HER2-positive gastric cancer patients from HER2-negative patients, suggesting its potential as a surrogate biomarker of HER2 status. Prospective studies are now needed to further validate our findings. In particular, the ability to predict patient prognosis and response by monitoring serum HER2 levels during disease progression and treatment warrants additional investigation.

## Supporting Information

S1 PRISMA Checklist (DOC)

## References

[1] Bang YJ, Van Cutsem E, Feyereislova A, Chung HC, Shen L, Sawaki A, et al Trastuzumab in combination with chemotherapy versus chemotherapy alone for treatment of HER2-positive advanced gastric or gastro-oesophageal junction cancer (ToGA): a phase 3, open-label, randomised controlled trial. Lancet. 2010;376(9742):687–97. 10.1016/S0140-6736(10)61121-X 20728210

[2] Fornaro L, Lucchesi M, Caparello C, Vasile E, Caponi S, Ginocchi L, et al Anti-HER agents in gastric cancer: from bench to bedside. Nature reviews Gastroenterology & hepatology. 2011;8(7):369–83. 21647199 10.1038/nrgastro.2011.81

[3] Ruschoff J, Hanna W, Bilous M, Hofmann M, Osamura RY, Penault-Llorca F, et al HER2 testing in gastric cancer: a practical approach. Modern pathology: an official journal of the United States and Canadian Academy of Pathology, Inc. 2012;25(5):637–50. 10.1038/modpathol.2011.198 22222640

[4] Dang HZ, Yu Y, Jiao SC. Prognosis of HER2 over-expressing gastric cancer patients with liver metastasis. World journal of gastroenterology: WJG. 2012;18(19):2402–7. 10.3748/wjg.v18.i19.2402 22654433 PMC3353376

[5] Gomez-Martin C, Garralda E, Echarri MJ, Ballesteros A, Arcediano A, Rodriguez-Peralto JL, et al HER2/neu testing for anti-HER2-based therapies in patients with unresectable and/or metastatic gastric cancer. Journal of clinical pathology. 2012;65(8):751–7. 10.1136/jclinpath-2012-200774 22569536 PMC3410298

[6] Janjigian YY, Werner D, Pauligk C, Steinmetz K, Kelsen DP, Jager E, et al Prognosis of metastatic gastric and gastroesophageal junction cancer by HER2 status: a European and USA International collaborative analysis. Annals of oncology: official journal of the European Society for Medical Oncology / ESMO. 2012;23(10):2656–62. 10.1093/annonc/mds104 22689179

[7] Allison M. The HER2 testing conundrum. Nature biotechnology. 2010;28(2):117–9. 10.1038/nbt0210-117 20139941

[8] Wolff AC, Hammond ME, Schwartz JN, Hagerty KL, Allred DC, Cote RJ, et al American Society of Clinical Oncology/College of American Pathologists guideline recommendations for human epidermal growth factor receptor 2 testing in breast cancer. Journal of clinical oncology: official journal of the American Society of Clinical Oncology. 2007;25(1):118–45. 17159189 10.1200/JCO.2006.09.2775

[9] Overman MJ, Modak J, Kopetz S, Murthy R, Yao JC, Hicks ME, et al Use of research biopsies in clinical trials: are risks and benefits adequately discussed? Journal of clinical oncology: official journal of the American Society of Clinical Oncology. 2013;31(1):17–22. 23129736 10.1200/JCO.2012.43.1718 PMC5545102

[10] Sui W, Ou M, Chen J, Wan Y, Peng H, Qi M, et al Comparison of immunohistochemistry (IHC) and fluorescence in situ hybridization (FISH) assessment for Her-2 status in breast cancer. World journal of surgical oncology. 2009;7:83 10.1186/1477-7819-7-83 19895711 PMC2776594

[11] Sheffield BS, Garratt J, Kalloger SE, Li-Chang HH, Torlakovic EE, Gilks CB, et al HER2/neu testing in gastric cancer by immunohistochemistry: assessment of interlaboratory variation. Archives of pathology & laboratory medicine. 2014;138(11):1495–502. 25357111 10.5858/arpa.2013-0604-OA

[12] Ludovini V, Gori S, Colozza M, Pistola L, Rulli E, Floriani I, et al Evaluation of serum HER2 extracellular domain in early breast cancer patients: correlation with clinicopathological parameters and survival. Annals of oncology: official journal of the European Society for Medical Oncology / ESMO. 2008;19(5):883–90. 10.1093/annonc/mdm585 18187484

[13] Farzadnia M, Meibodi NT, Shandiz FH, Mahmoudi M, Bahar MM, Memar B, et al Evaluation of HER2/neu oncoprotein in serum and tissue samples of women with breast cancer: correlation with clinicopathological parameters. Breast. 2010;19(6):489–92. 10.1016/j.breast.2010.05.012 20675140

[14] Sugano K, Ushiama M, Fukutomi T, Tsuda H, Kitoh T, Ohkura H. Combined measurement of the c-erbB-2 protein in breast carcinoma tissues and sera is useful as a sensitive tumor marker for monitoring tumor relapse. International journal of cancer Journal international du cancer. 2000;89(4):329–36. 10956406 10.1002/1097-0215(20000720)89:4<329::aid-ijc3>3.0.co;2-p

[15] Sasaki T, Fuse N, Kuwata T, Nomura S, Kaneko K, Doi T, et al Serum HER2 levels and HER2 status in tumor cells in advanced gastric cancer patients. Japanese journal of clinical oncology. 2015;45(1):43–8. 10.1093/jjco/hyu174 25378649

[16] Dai SQ, An X, Wang F, Shao Q, Chen YC, Kong YN, et al Serum HER 2 Extracellular Domain Level Is Correlated with Tissue HER 2 Status in Metastatic Gastric or Gastro-Oesophageal Junction Adenocarcinoma. PloS one. 2013;8(5). 10.1371/journal.pone.0063458 PMC3653938 23691049

[17] Li X, Tu J, Zhang D, Xu Z, Yang G, Gong L, et al The clinical significance of HER-2 and NF-KB expression in gastric cancer. Hepato-gastroenterology. 2013;60(126):1519–23. 23933945 10.5754/hge13242

[18] Oyama K, Fushida S, Tsukada T, Kinoshita J, Watanabe T, Shoji M, et al Evaluation of serum HER2-ECD levels in patients with gastric cancer. Journal of gastroenterology. 2015;50(1):41–5. 10.1007/s00535-014-0941-3 24557054

[19] Leeflang MM, Deeks JJ, Gatsonis C, Bossuyt PM, Cochrane Diagnostic Test Accuracy Working G. Systematic reviews of diagnostic test accuracy. Annals of internal medicine. 2008;149(12):889–97. 19075208 10.7326/0003-4819-149-12-200812160-00008 PMC2956514

[20] Moher D, Liberati A, Tetzlaff J, Altman DG, Group P. Preferred reporting items for systematic reviews and meta-analyses: the PRISMA statement. PLoS medicine. 2009;6(7):e1000097 10.1371/journal.pmed.1000097 19621072 PMC2707599

[21] Peng Z, Liu Y, Li Y, Zhang X, Zhou J, Lu M, et al Serum HER2 extracellular domain as a potential alternative for tissue HER2 status in metastatic gastric cancer patients. Biomarkers in medicine. 2014;8(5):663–70. 10.2217/bmm.14.10 25123035

[22] Whiting PF, Rutjes AW, Westwood ME, Mallett S, Deeks JJ, Reitsma JB, et al QUADAS-2: a revised tool for the quality assessment of diagnostic accuracy studies. Annals of internal medicine. 2011;155(8):529–36. 10.7326/0003-4819-155-8-201110180-00009 22007046

[23] Reitsma JB, Glas AS, Rutjes AW, Scholten RJ, Bossuyt PM, Zwinderman AH. Bivariate analysis of sensitivity and specificity produces informative summary measures in diagnostic reviews. Journal of clinical epidemiology. 2005;58(10):982–90. 16168343 10.1016/j.jclinepi.2005.02.022

[24] Jaeschke R, Guyatt GH, Sackett DL. Users' guides to the medical literature. III. How to use an article about a diagnostic test. B. What are the results and will they help me in caring for my patients? The Evidence-Based Medicine Working Group. Jama. 1994;271(9):703–7. 8309035 10.1001/jama.271.9.703

[25] Fischer JE, Bachmann LM, Jaeschke R. A readers' guide to the interpretation of diagnostic test properties: clinical example of sepsis. Intensive care medicine. 2003;29(7):1043–51. 12734652 10.1007/s00134-003-1761-8

[26] Glas AS, Lijmer JG, Prins MH, Bonsel GJ, Bossuyt PM. The diagnostic odds ratio: a single indicator of test performance. Journal of clinical epidemiology. 2003;56(11):1129–35. 14615004 10.1016/s0895-4356(03)00177-x

[27] Higgins JP, Thompson SG, Deeks JJ, Altman DG. Measuring inconsistency in meta-analyses. Bmj. 2003;327(7414):557–60. 12958120 10.1136/bmj.327.7414.557 PMC192859

[28] Cen D, Xu L. Differential diagnosis between malignant and benign breast lesions using single-voxel proton MRS: a meta-analysis. Journal of cancer research and clinical oncology. 2014;140(6):993–1001. 10.1007/s00432-014-1605-7 24595596 PMC11823543

[29] Gan Y, Liang Q, Song X. Diagnostic value of alpha-L-fucosidase for hepatocellular carcinoma: a meta-analysis. Tumour biology: the journal of the International Society for Oncodevelopmental Biology and Medicine. 2014;35(5):3953–60. 24395655 10.1007/s13277-013-1563-8

[30] Zhu X, Lv M, Wang H, Guan W. Identification of circulating microRNAs as novel potential biomarkers for gastric cancer detection: a systematic review and meta-analysis. Digestive diseases and sciences. 2014;59(5):911–9. 10.1007/s10620-013-2970-9 24337687

[31] Deeks JJ, Macaskill P, Irwig L. The performance of tests of publication bias and other sample size effects in systematic reviews of diagnostic test accuracy was assessed. Journal of clinical epidemiology. 2005;58(9):882–93. 16085191 10.1016/j.jclinepi.2005.01.016

[32] Takahashi Y, Ohori H, Takahashi M, Gamoh M. Serum HER2 extracellular domain levels and tissue HER2 overexpression in advanced gastric cancer patients. Annals of Oncology. 2013;24:ix31.

[33] Takahashi M, Takahashi Y, Ohori H, Gamoh M. Serum HER2ECD levels in patients with advanced gastric cancer treated with trastuzumab as first-line therapy. Annals of Oncology. 2013;24:ix92.

[34] Saito M, Kaneto H, Okuda H, Kodaira J, Hagiwara T, Kozawa H, et al Serum HER2 in gastric cancer. Annals of Oncology. 2013;24:ix31.

[35] An X, Dai S, Wang F, Shao Q, Chen C, Chen Y, et al Evaluation of serum HER2 extracellular domain in in metastatic gastric or gastroesophageal junction cancer: Correlation with HER2 status by immunohistochemistry and fluorescence in situ hybridization and clinicopathologic parameters. Journal of Clinical Oncology. 2013;31(15).

[36] Sasaki T, Fuse N, Kuwata T, Takahashi M, Asano H, Kaneko K, et al Relationship between serum HER2 level and histologic HER2 status in patients with advanced/recurrent gastric cancer. Journal of Clinical Oncology. 2012;30(4).

[37] Park KU, Lee HE, Nam SK, Nam KH, Park DJ, Kim HH, et al The quantification of HER2 and MYC gene fragments in cell-free plasma as putative biomarkers for gastric cancer diagnosis. Clinical Chemistry and Laboratory Medicine. 2014;52(7):1033–40. 10.1515/cclm-2013-0988 24670359

[38] Tsigris C, Karayiannakis AJ, Syrigos KN, Zbar A, Diamantis T, Kalahanis N, et al Clinical significance of soluble c-erbB-2 levels in the serum and urine of patients with gastric cancer. Anticancer research. 2002;22(5):3061–5. 12530043

[39] Kono K, Naganuma H, Sekikawa T, Amemiya H, Takahashi A, Iizuka H, et al Serum level of HER-2/neu in patients with gastric cancer: correlation with HER-2/neu overexpression in gastric carcinoma tissue. Tumour biology: the journal of the International Society for Oncodevelopmental Biology and Medicine. 2000;21(3):139–44. 10754464 10.1159/000030120

[40] Narita T, Seshimo A, Suzuki M, Murata J, Kameoka S. Status of tissue expression and serum levels of HER2 in gastric cancer patients in Japan. Hepato-gastroenterology. 2013;60(125):1083–8. 10.5754/hge121022 23321006

[41] Takehana T, Kunitomo K, Kono K, Kitahara F, Iizuka H, Matsumoto Y, et al Status of c-erbB-2 in gastric adenocarcinoma: a comparative study of immunohistochemistry, fluorescence in situ hybridization and enzyme-linked immuno-sorbent assay. International journal of cancer Journal international du cancer. 2002;98(6):833–7. 11948459 10.1002/ijc.10257

[42] Albarello L, Pecciarini L, Doglioni C. HER2 testing in gastric cancer. Advances in anatomic pathology. 2011;18(1):53–9. 10.1097/PAP.0b013e3182026d72 21169738

[43] Witzel I, Loibl S, von Minckwitz G, Mundhenke C, Huober J, Hanusch C, et al Monitoring serum HER2 levels during neoadjuvant trastuzumab treatment within the GeparQuattro trial. Breast cancer research and treatment. 2010;123(2):437–45. 10.1007/s10549-010-1030-9 20623180

[44] Swets JA. Measuring the accuracy of diagnostic systems. Science. 1988;240(4857):1285–93. 3287615 10.1126/science.3287615

[45] Fagan TJ. Letter: Nomogram for Bayes theorem. The New England journal of medicine. 1975;293(5):257 1143310 10.1056/NEJM197507312930513

[46] Kong SY, Kang JH, Kwon Y, Kang HS, Chung KW, Kang SH, et al Serum HER-2 concentration in patients with primary breast cancer. Journal of clinical pathology. 2006;59(4):373–6. 16461567 10.1136/jcp.2005.029603 PMC1860357

